# Linear Elastic Fracture Mechanics Characterization of an Anisotropic Shale

**DOI:** 10.1038/s41598-018-26846-y

**Published:** 2018-05-31

**Authors:** Y. Luo, H. P. Xie, L. Ren, R. Zhang, C. B. Li, C. Gao

**Affiliations:** 1 Key Laboratory of Deep Underground Science and Engineering (MOE), College of Architecture and Environment, Sichuan University, Chengdu, 610065 China; 2 Institute of Deep Earth Science and Green Energy, Shenzhen University, Shenzhen, 518060 China; 3 College of Water Resources and Hydropower, Sichuan University, Chengdu, 610065 China

## Abstract

The existence of bedding planes in natural shale formations makes the fracture characterization remarkably complicated. To achieve a further understanding of the anisotropic crack extension behaviors of shale using a linear elastic fracture mechanics approach, four groups of three-point bending tests on Longmaxi shale from southeast Chongqing were conducted in this study with different bedding plane inclination angles. The fracture propagation paths were observed using a scanning electron microscope. The results indicated that cracks initiated along the bedding plane when the bedding plane inclination angle (i.e., the angle between the loading direction and the normal direction of the bedding plane) was relatively large; in contrast, cracks penetrated into the matrix and induced higher fracture toughness in cases with lower bedding plane inclination angle. Brittle fractures occurred in the tested shale, and the fracture strength was strongly dependent on the bedding plane inclination angle. Meanwhile, the stress field around the crack tip was analyzed theoretically. The results indicated that the crack tip stress field of anisotropic shale is not only determined by the stress intensity factor but also related to the elastic constants and bedding plane inclination angle. Furthermore, a criterion for determining whether a crack extends along the bedding plane was developed by distinguishing the differences in the strengths of the shale bedding and the matrix.

## Introduction

Over the past few decades, hydraulic fracturing has been widely applied in low-permeability shale reservoir stimulations around the world^1,2^. Such fracturing involves some fundamental mechanical processes, including the crack initiation and propagation in shale rock^3,4^, which are dependent on the *in-situ* stress field, pore pressure, hydraulic load of the fracturing liquid, and the mechanical properties of the reservoir rock^5^. Deformation and crack growth in the reservoir significantly affect the flow of the fracturing liquid inside the rough-walled fractures^3^. Fracture mechanics, which mainly addresses crack initiation, propagation and arrest, is suitable for hydraulic fracturing studies^6–9^. Nevertheless, the fracture extension mechanism of anisotropic shale remains far from being clearly understood.

The fracture toughness, *K*_I*c*_, which characterizes the ability of a material to resist crack propagation, is a key mechanical parameter that controls hydraulic fracture propagation^7,10^. In the numerical models developed using a linear elastic fracture mechanics (LEFM) approach, a criterion that states *K*_I_ = *K*_I*c*_, where *K*_I_ is the mode I stress intensity factor (SIF), has been widely employed to determine the hydraulic fracture propagation condition^11–13^. To employ such an approach in shale reservoir stimulation simulations, the fracture toughness should be tested first. The existing shale fracture mechanics tests (Schmidt and Huddle, 1977; Lee, *et al*., 2015; Chandler *et al*., 2016)^14–16^ show that the fracture toughness on shales are highly anisotropic, and the measured results are scattered between different shales and even for the same type of shale owing to (1) the inhomogeneity of shales, (2) the elastic anisotropy nature of shale matrix, (3) strong non-elastic deformation, and (4) the strong environmental effect^17^, e.g., humidity, in influencing the testing results. Notably, that although some fracture toughness tests for shale have been conducted, available experimental data on the fracture toughness of shale remain relatively sparse, and some test data were not correctly calculated due to the failure to account for of layers. More importantly, the effect of the bedding orientation on the fracture toughness was not adequately evaluated and requires a further investigation. Aiming at this, in the current work, the fracture toughness of four groups of dry Longmaxi shale from southeast Chongqing, China, was tested using the notched deep beam (NDB) configuration proposed by Luo, *et al*.^18^. The anisotropic characteristics of the fracture toughness of the Longmaxi shale were discussed at the laboratory scale. The specimens contained four different bedding plane orientation angles, which were defined as the angle between the normal direction of the bedding plane and the loading direction, i.e., *β* = 0°, 30°, 60° and 90°. It should be mentioned that the effect of the hydraulic liquid on the fracture properties of the tested shale is beyond the main consideration of this work and is not discussed.

In addition to the fracture toughness, the fracture paths are also important for understanding the fracture mechanism of shale. During the shale fracking process, the bedding plane usually acts as a plane of weakness that diverts crack propagation, i.e., the fractures can either extend along or penetrate across the bedding plane when a fracture meets a bedding plane^19^. By investigating crack propagation trajectories in SCB specimens made of shale containing calcite-filled veins, Lee, *et al*.^15^ found that the fracture propagation was more likely to divert into the vein when the angle between the vein plane and pre-existing crack was more oblique. However, they did not quantitatively consider the effect of the angle between the original crack and the shale bedding plane on the fracture propagation direction. With the development of experimental technology, *in situ* scanning electron microscopy (SEM) with loading capability was employed to study the fracture behavior of rock materials^20–23^. For instance, Zuo, *et al*.^22^ investigated the effect of elevated temperature on the grain boundary cracking behavior and intergranular cracking behavior of sandstone using an SEM. In the current work, SEM observations of shale fracture propagation paths were conducted during the aforementioned three-point bending tests, and the results were used to further reveal the anisotropic fracture process in shale.

Theoretically, to better understand the competing processes (propagation along or across the bedding) in shale, an analysis of the stress field surrounding the crack tip was desirable. In the LEFM, a singular stress field has been widely adopted to analyze the strength of cracked solid bodies made of brittle media^24–26^. Considering the parallel bedding distribution, shale has always been considered as a typical transversely isotropic material^27,28^. The elastic anisotropy of rocks has a significant influence on the crack propagation^29^. A transversely isotropic body with a crack that is not associated with the elastic symmetry plane may be treated as a crack problem in generally anisotropic bodies^30^. The stress concentration existing in crack tips that is distinct from that of an isotropic body deserves serious consideration. Sih *et al*.^30^ derived the analytical solution of the singular stress field surrounding a crack tip in an orthotropic body. Heng *et al*.^19^ applied Sih’s solution to study the stress fields of a cracked shale body and confirmed that the stress fields around a crack tip in anisotropic materials depended on not only the stress intensity factor (SIF) but also the elastic constants. However, Heng, *et al*. did not analyze the strength and failure modes of the cracked shale based on the stress field. In the current work, the stress field around a crack tip in anisotropic shale is analyzed in depth, and the conclusions are applied to quantitatively study the failure modes of shale by considering the differences of strength between the shale bedding and matrix.

## Rock Properties and Testing Method

### Fundamental properties of the investigated shale

**Figure 1:**
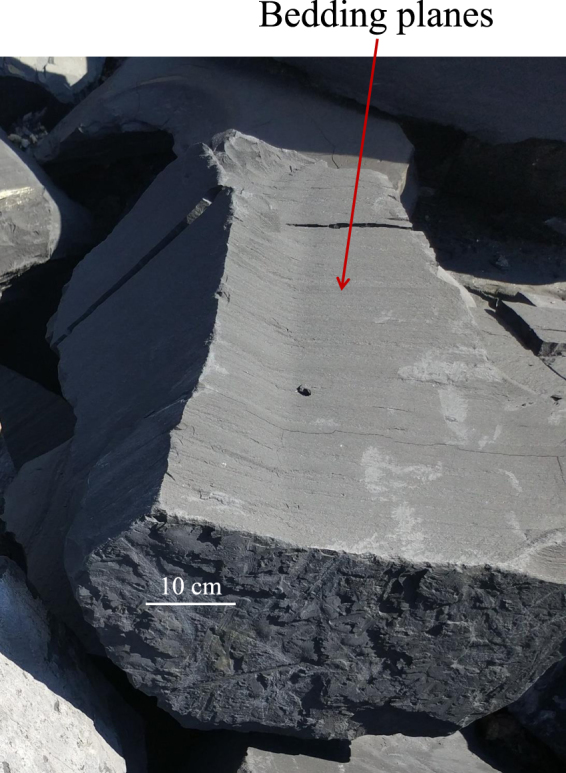
Longmaxi shale outcrops with visible bedding planes.

The tested shale was taken from outcrops of the Longmaxi Formation deposited during the Silurian period in Chongqing, Southwest China. Its dry density is approximately 2.66 g/cm^3^. The mineral species and contents in the tested shale were obtained via X-ray diffraction (XRD) analysis (see Table 1), which indicated that the shale is mainly composed of clay minerals, quartz, albite, etc. Based on mercury intrusion porosimetry and nuclear magnetic resonance, He, *et al*.^31^ demonstrated that the pore throat radius of the shale in this area is 6–15 nm and the porosity is 4.7–5.2%. The outcrop is a dull brown carbonaceous shale, which comprise laminations of alternating matrix and bedding according to visual inspection. The bedding planes in the shale blocks are clearly visible in Fig. 1. Based on micro-scale image processing of thin sections, Zhang, *et al*.^32^ found that most layers of Longmaxi shale are narrow with 42.31% of the inclusion having a thickness in the range of [1 *μ*m, 10 *μ*m] and 63.81% of the matrix having a thickness in the range of [1 *μ*m, 53 *μ*m], and the mean particle grain diameters are about 5–15 *μ*m. As a typical sedimentary rock, the Longmaxi shale can be regarded as a typical transversely isotropic material, with a symmetry axis perpendicular to the sedimentary plane^27,28^. According to a series of uniaxial unconfined compression tests on the shale with different bedding plane orientations, the five elastic constants, i.e., *E*_1_, *E*_2_, *v*_12_, *v*_23_ and *G*_12_, were determined to be 18.799 GPa, 24.344 GPa, 0.229, 0.166, and 8.433 GPa, respectively. Herein, the shear modulus *G*_12_ was calculated using the Saint-Venant’s formula^33^.

**Table 1:** Minerals content of the tested shale.

Mineral types	Quartz	Albite	Calcite	Dolomite	Potash feldspar	Pyrite	Clay minerals
Mass percentage (%)	37.0	10.9	7.7	5.1	3.2	1.9	34.2

### Sample preparation and testing procedure

**Figure 2:**
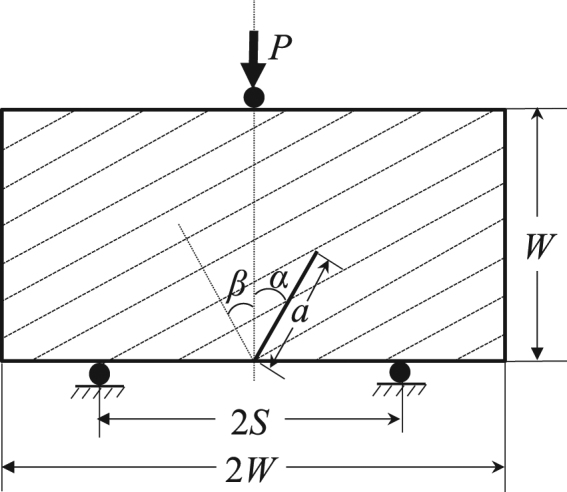
NDB specimen (the dashed lines represent the shale bedding direction).

The test configuration selected in this study was a notched deep beam (NDB) specimen^18^, which is a brick-type rectangle with a length to width ratio (*L*/*W*) of 2.0 and contains an edge crack of length *a*. The NDB specimen was loaded by a vertical load *P* under symmetrical three-point bending. The distance between the bottom supports of the loading fixture was 2*S* (see Fig. 2). NDB specimens were chosen for this research because these specimens can be easily obtained by simply cutting them from the shale block, and the required loading device is also simple. The NDB samples are able to provide pure mode I, pure mode II, and any intermediate mixed-mode loading conditions by employing different combinations of the crack length *a*, crack inclined angle 𝛼 relative to the loading direction and half span distance *S*. For isotropic rock, when the inclined crack angle 𝛼 is zero, the specimen is subjected to pure mode I loading. By increasing 𝛼, mode II deformation is intensified. Pure mode II fracture (*Y*_I_ = 0) occurs at a specific angle 𝛼 depending on the crack length ratio *a*/*W* and loading span ratio *S*/*W*^18^.

The selected sizes of shale NDB specimens were 20 mm long (i.e., *L* = 20 mm), 10 mm wide (i.e., *W* = 10 mm) and 5 mm thick (i.e., *B* = 5 mm). The values of *S*/*W* and *a*/*W* were both set to 0.5 for all specimens. The pre-crack length *a* = 5 mm and the inclined crack angle 𝛼 = 0° were employed to test the fracture toughness of the shale in this study. As illustrated in Fig. 2, β denotes the bedding plane inclination angle, which is defined as the angle between the original crack and the normal direction of the bedding plane and ranges between 0° and 90°. Four different values of *β*, i.e., *β* = 0°, 30°, 60° and 90°, were selected. Four samples were manufactured for each bedding plane inclination angle, yielding a total of 16 shale NDB specimens. In order to prevent shale swelling with water, the specimens were machined by dry cutting. The edge cracks in the NDB specimens were prepared using a 0.2 mm thick diamond impregnated wire saw. To observe the crack propagation path during the experiments using SEM, after the notch cutting, the surfaces of each specimen were coated with a layer of gold film with a thickness of 5–10 nm.

**Figure 3:**
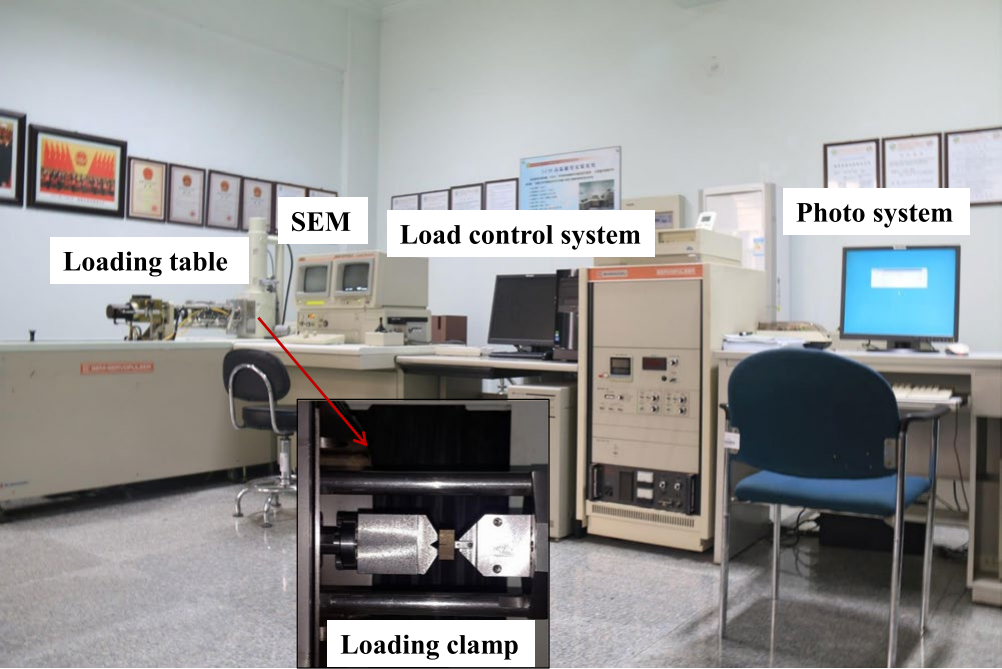
Configuration of the digital hydraulic test system and SEM.

**Figure 4:**
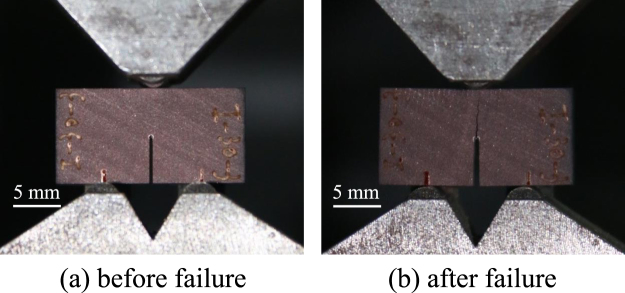
Shale sample and three-point bending clamp (taking *β* = 30° as an example).

The fracture tests of the Longmaxi shale were performed using an advanced digital hydraulic mechanical testing apparatus with an *in situ* SEM scanning system located at the China University of Mining and Technology. During testing, SEM images of the specimens were acquired in real time. Figure 3 shows the details of the apparatus, which is composed of one loading system and one SEM system. Each NDB specimen was placed inside a three-point bending fixture (see Fig. 4), and then the fixture was positioned inside the SEM chamber under vacuum. The observation area of the samples corresponded to the SEM image field, which focused on the same position around the original crack tip. All the specimens were tested at room temperature. The loading rate for all experiments was 0.05 mm/min. Meanwhile, the SEM system was employed to continuously record the crack propagation paths during the loading process, which occurred in the vacuum chamber.

## Experimental Results

### Fracture toughness of the anisotropic shale for various bedding plane inclination angles

**Figure 5:**
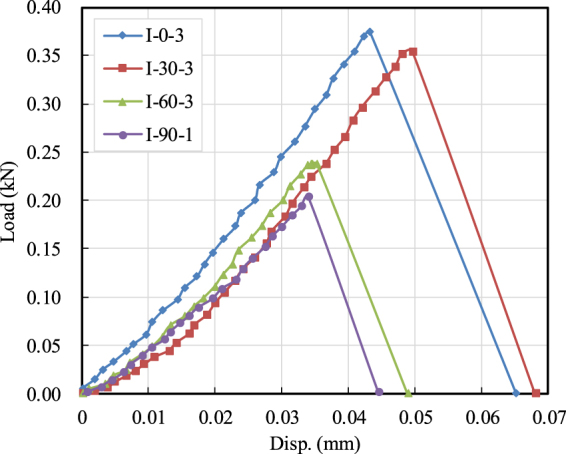
Load-displacement curves of the shale specimens with different bedding plane inclination angles.

Figure 5 shows typical plots of the measured load versus the loading-point displacement for different bedding plane inclination angles. All of the load-displacement curves are relatively gentle during the initial loading stages. This gentle trend is induced by adjusting the contact between the specimen and the pressure head of the testing machine. The later stages exhibit approximately linear growth. For all the NDB specimens, when the loads reach the peak values, the load-displacement curves drop instantaneously and exhibit a straight line because only one post-peak data point was recorded by the mechanical testing system. Thus, the post-peak curves plotted in Fig. 5 do not represent the true material dynamic response. At the same time, the specimens fail and split into two pieces, as illustrated in Fig. 4(b). The crack propagation speed was so rapid that the SEM scanning system could not record transitional images. This load-displacement curve trend indicates typical brittle fracture behavior.

The mode I and mode II SIFs *K*_I_ and *K*_II_ of isotropic materials in the NDB specimen can be written as functions of the geometric parameters and the applied load^18^. However, the SIFs for an anisotropic material, such as shale, are related not only to the elastic constants of the material but also to the bedding layer orientation^34^, so the functions can be written as follows:

1 $${K}_{{\rm{{\rm I}}}}=\frac{P\sqrt{{\rm{\pi }}a}}{2WB}{Y}_{{\rm{I}}}(\frac{a}{W},\frac{S}{W},{\alpha },{{E}}_{1},{{E}}_{2},{{v}}_{12},{{v}}_{23},{{G}}_{12},{\beta }),$$

2 $${K}_{{\rm{II}}}=\frac{P\sqrt{{\rm{\pi }}a}}{2WB}{Y}_{{\rm{II}}}(\frac{a}{W},\frac{S}{W},{\alpha },{{E}}_{1},{{E}}_{2},{{v}}_{12},{{v}}_{23},{{G}}_{12},{\beta }).$$

where *P* is the applied load; *B* is the thickness of the specimen; *Y*_I_ and *Y*_II_ are the mode I and mode II dimensionless SIFs, respectively, which can be calculated using numerical methods.

**Figure 6:**
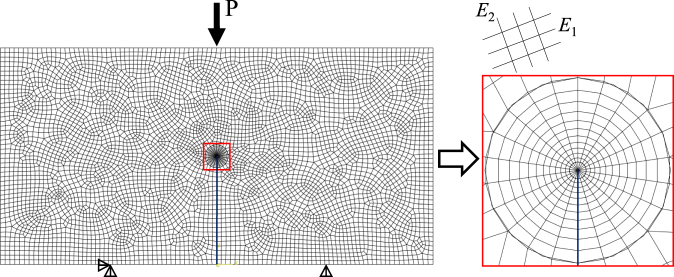
The finite element mesh for an NDB specimen and a close view of the mesh near the crack tip.

For a given configuration, the numerical values of *K*_I_ and *K*_II_ can be calculated using the finite element model (FEM) code ABAQUS, which calculates the SIFs based on the *J*-integral of orthotropic materials^35^. The non-dimensional parameters, *Y*_I_ and *Y*_II_, of this NDB specimen for the anisotropic shale can then be obtained from Eqs (1) and (2). Several finite element models were established to calculate the non-dimensional parameters, *Y*_I_ and *Y*_II_, for the NDB configuration of the anisotropic shale using ABAQUS. The anisotropic elastic constants and geometry parameters adopted in the numerical model are listed in Table 2, and the material was assumed to behave in a linear elastic manner. In the current numerical simulations, approximately 5800 8-node biquadratic plane strain quadrilateral elements (CPE8) were meshed, as shown in Fig. 6. The displacement in the Y direction was set to zero for the two bottom supports, and the displacement in the X direction for the left bottom support was also set to zero. A reference load *P* was applied at the upper loading point. Meanwhile, the different orientations of bedding planes relative to the notch were determined while changing the material orientation of the finite element model. The values of *Y*_I_ and *Y*_II_ for different bedding layer orientations (*β* = 0°, 30°, 60° and 90°) are summarized in Table 3. The variations of *Y*_I_ and *Y*_II_ for different *β* angles further proved that for the anisotropic material, the dimensionless SIFs should also depend on the orientation of the bedding planes relative to the notch. Compared with *Y*_I_, *Y*_II_ is relatively small (the maximum *Y*_II_/*Y*_I_ percentage is less than 5% for all cases). Therefore, the influence of the mode II SIF on the fracture strength is negligible for the tested shale, and only the mode I SIF was considered in this work.

**Table 2:** The material properties and geometry parameters selected in the finite element model of the NDB samples.

Parameter	Value
*E*_1_ (GPa)	18.799
*E*_2_ (GPa)	24.344
*v*_12_	0.229
*v*_23_	0.166
*G*_12_ (GPa)	8.433
*W* (mm)	10
*S* (mm)	5
*a* (mm)	5
*B* (mm)	5

**Table 3:** The non-dimensional parameters of the NDB samples for different shale bedding plane inclination angles *β*.

*β* (°)	*Y*_I_	*Y*_II_	*Y*_II_/*Y*_I_ (%)
0	3.386	0.000	0.000
30	3.421	0.127	3.708
60	3.475	0.165	4.735
90	3.495	0.000	0.000

**Figure 7:**
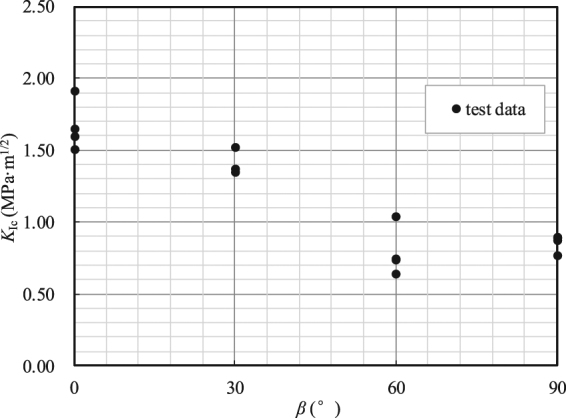
Variation of fracture toughness *K*_Ic_ with the shale bedding plane inclination angles *β*.

The peak loads *P*_*cr*_ measured during the shale fracture tests are tabulated in Table 4, except for that of specimen I-30-4, which was destroyed during preloading because of an operating error. If a LEFM approach is taken and ideally brittle samples are inherently assumed, the critical SIF, namely, the fracture toughness *K*_I*c*_, of the tested shale can be calculated using Eq. (1) by substituting the measured peak load *P*_*cr*_ for the applied load *P* and the non-dimensional parameter *Y*_I_ in Table 3. Figure 7 shows the variation of the fracture toughness *K*_I*c*_ with the shale bedding plane inclination angle *β*. The values of *K*_I*c*_ when *β* = 60° and 90° are substantially lower than those for the other two angles. The tested values of *K*_I*c*_ vary from 0.6 to 1.9 MPa·m^1/2^. The obtained fracture toughness is slightly higher than those obtained by Heng, *et al*.^19^ for Longmaxi shale: 1.146 MPa·m^1/2^ when the crack plane was normal to the bedding plane and 0.566 MPa·m^1/2^ when the crack plane was parallel to the bedding plane. The reasons could be (1) the influence of the thickness of the preexisting notch, and (2) the vacuum dried conditions for the shale fracture tests, as rocks with lower water contents are usually stronger^36,37^. The fracture toughness anisotropy ratio between the maximum *K*_I*c*_ and minimum *K*_I*c*_ is approximately 2.12, which is approximately equal to the experimental result in Heng, *et al*.^19^. This result clearly demonstrates that the fracture strength anisotropy of the shale depends on the bedding plane orientation and is significant.

**Table 4:** Experimental results of the fracture tests on the shale NDB specimens.

Specimen code	*β* (°)	*P*_cr_ (kN)	*K*_IC_ (MPa · m^1/2^)
I-0-1	0	0.3879	1.646
I-0-2	0	0.3544	1.504
I-0-3	0	0.3748	1.591
I-0-4	0	0.449	1.905
I-30-1	30	0.3134	1.344
I-30-2	30	0.3179	1.363
I-30-3	30	0.3543	1.519
I-60-1	60	0.1463	0.637
I-60-2	60	0.1681	0.732
I-60-3	60	0.2383	1.038
I-60-4	60	0.1698	0.740
I-90-1	90	0.2043	0.895
I-90-2	90	0.1978	0.866
I-90-3	90	0.1739	0.762
I-90-4	90	0.2011	0.881

### Meso-fracture propagation paths for various bedding plane inclination angles

**Figure 8:**
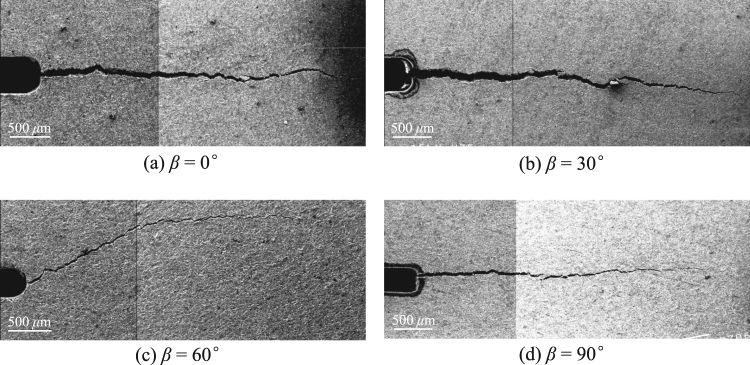
Typical meso-fracture propagation paths of the shale samples with different bedding plane inclination angles obtained from an SEM (scale bar 500 *μ*m).

Figure 8 shows SEM images of the meso-fracture propagation paths of the shale samples with different bedding plane inclination angles. For a particular bedding plane inclination angle, although the peak loads have some discreteness, the observed fracture propagation paths for the samples with the same *β* are basically consistent. The fracture test results indicate that the bedding plane orientation has a significant influence on the failure modes of the anisotropic shale. When *β* = 0°, 30° and 90°, the fracture initiated at the original crack tip and propagated straight along the original crack line. These crack propagation paths are rather straight, which is consistent with the pure mode I fracture growth path of isotropic rocks^38^. The modeling results by Fan *et al*.^29,39^ and the field observations^40^ are consistent with the experimental results that fracture growth behavior accord with those in isotropic rocks when the original crack is aligned with the shale bedding direction. Nevertheless, compared to the other angles, when *β* = 60°, the fracture propagation path is significantly different in the images. When the bedding plane inclination angle is 60°, the crack also initiated at the original crack tip, but this path deviated from the original crack line and propagated along the bedding plane direction. After a short extension, the crack turned to parallel to loading direction, i.e., the crack initiation angle became 30° with respect to the original crack direction rather than exhibiting self-similar fracture propagation. In terms of the shale failure mechanism, the self-similar crack propagations when *β* = 0° and 30° are mainly associated with matrix tensile failure, whereas the damage at *β* = 90° corresponds to bedding plane tensile fracture. The failure modes at *β* = 60° are attributed to the tensile-shear stress existing in both the matrix and the bedding. Therefore, it could be supposed that the traditional isotropic LEFM theory no longer works for anisotropic shale. In theory, how to identify the crack propagation trajectory for layered shale with strong anisotropy remains a significant challenge.

Based on the aforementioned analyses, we determined that the bedding plane orientation greatly affects the characteristics of shale fracture mechanics, impacting not only the fracture toughness but also the meso-fracture propagation paths. It can be concluded that the crack will initiate along the weak bedding plane when the bedding plane inclination angles are relatively large (*β* = 60° and 90°), while it will penetrate into the matrix when the bedding plane inclination angles are smaller (*β* = 0° and 30°). The energy required to propagate fractures along the bedding surface is clearly far less than that in the matrix, leading to the ability to resist crack propagation, i.e., the fracture toughness, as *β* = 60° and 90° are considerably lower than those of the other two cases. The induced fracture becomes more likely to divert into the weak bedding as the bedding plane inclination angle becomes larger.

## Theoretical Analysis of the Anisotropic Shale with a Crack Based on LEFM

### Crack tip stress field of the anisotropic shale

**Figure 9:**
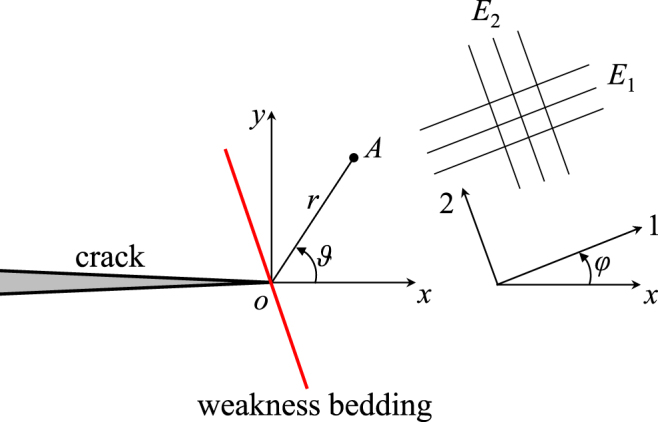
Local coordinate system of the stress field near the crack tip in the direction perpendicular to the shale bedding plane (the red line represents the shale bedding).

Shale is regarded as a transversely isotropic material with a symmetry axis perpendicular to the bedding planes. Figure 9 defines the local *x-o-y* coordinate system in the plane normal to the shale bedding. The origin is defined at the crack tip, and the *x*-axis is along the original crack direction. The coordinate system (1, 2) corresponds to the material principal axes. As shown in Fig. 9, the angle between a principal direction of the shale and the *x*-axis is defined as *φ* (equivalent to the angle *β* in Fig. 2). For plane symmetric loading, the tangential stress in polar coordinates (*r*, *ϑ*) around a crack tip can be written as follows:

3 $${{\sigma }}_{{\vartheta }{\vartheta }}=\frac{{K}_{{\rm{I}}}}{\sqrt{2{\pi }r}}{{\sigma }}^{\ast }({\vartheta })={{\sigma }}_{xx}\,{\sin }^{2}{\vartheta }+{{\sigma }}_{yy}\,{\cos }^{2}{\vartheta }-{{\tau }}_{xy}\,\sin (2{\vartheta }),$$

where *r* and *ϑ* are the polar coordinates of point *A* (Fig. 9); *K*_I_ is the mode I SIF; *σ*_*xx*_, *σ*_*yy*_ and *τ*_*xy*_ are the singular stresses at point *A* in the local Cartesian coordinate system (see Appendix for details). The parameter σ*, i.e., the dimensionless tangential stress, can be calculated using the following equations:

4 $${{\sigma }}^{\ast }({\vartheta })={\rm{Re}}[\frac{{{\mu }}_{1}}{{{\mu }}_{1}-{{\mu }}_{2}}{(\cos {\vartheta }+{\mu }_{2}\sin {\vartheta })}^{3/2}-\frac{{{\mu }}_{2}}{{{\mu }}_{1}-{{\mu }}_{2}}{(\cos {\vartheta }+{\mu }_{1}\sin {\vartheta })}^{3/2}],$$

in which Re denotes taking the real part of the complex function; *μ*_1_ and *μ*_2_ are given in the Appendix. The dimensionless tangential stress σ* directly reflects the tangential stress level.

**Figure 10:**
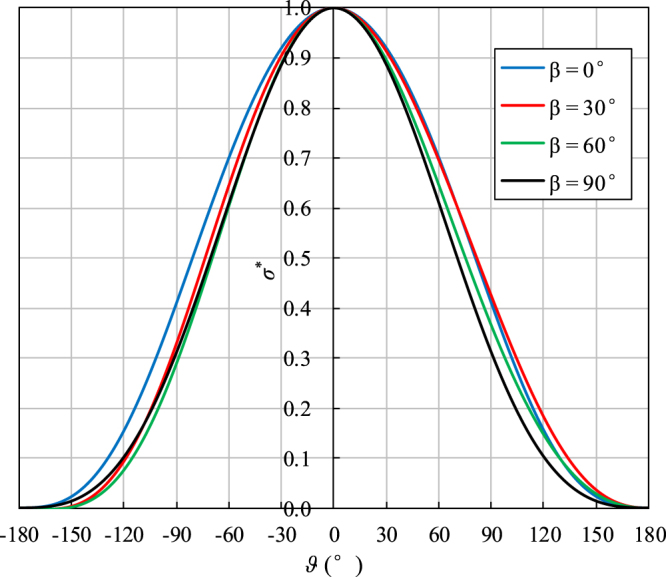
Variations of σ* with the polar coordinate angles *ϑ* for the four bedding plane inclination angles.

Setting the bedding plane direction angle *β* as 0°, 30°, 60° and 90°, the dimensionless characteristic tangential stress σ* can be calculated using Eq. (4). Figure 10 shows the variations of σ* with the polar coordinate angle *ϑ* for the plane strain case. As seen from the curves, σ* decreases from 1 to 0 as the angle *ϑ* gradually varies from 0° to ±180° for all bedding plane inclination angles. For *β* = 0° and 90°, the curves are symmetric about the *y*-axis, whereas the curves for *β* = 30° and 60° are not axisymmetric. This trend occurs because the bedding planes are symmetric about the original crack line when *β* = 0° and 90°; however, for other specimens, the original crack lines are not the axis of symmetry of the bedding planes. Thus, the stress field distribution of anisotropic shale near the crack tip is not only determined by the SIF but also related to the elastic constants and bedding plane inclination angles. Figure 10 also illustrates that there is no notable difference in the tangential stress distribution among the four bedding plane direction angles. This high degree of similarity occurs because the difference between the elastic parameters in the two principal directions of the tested shale (i.e., *E*_1_, *E*_2_, *v*_12_ and *v*_23_) is small.

### Criterion of the crack propagation along the bedding plane

A significant open issue should be discussed, namely, how the weak bedding surfaces interact with crack propagation in shale. Zeng and Wei^41^ extended the energy-based crack deflection criterion under the influence of a weak shale interface. Whereas the shale was considered to be an isotropic medium in that study, which does not truly reflect the mechanical behavior of shale because the bedding plane inclination angle also influences the stress field. According to the maximum tangential stress (MTS) criterion^25^, which considers that a crack will propagate along the direction in which the tangential stress *σ*_*ϑϑ*_ is the maximum and that the crack initiation will occur once the maximum *σ*_*ϑϑ*_ reaches a critical value *σ*_*ϑϑc*_, the fracture in all specimens in this test will propagate straight along the direction *ϑ* = 0°, i.e., the original crack line. The reason is that for various *β* values, the tangential stress reaches a maximum as *ϑ* = 0°, as illustrated in Fig. 10. Nevertheless, the MTS criterion does not correspond to the experimental results for *β* = 60°. The tested shale at the microscopic scale is considered as the rock matrix containing a set of parallel bedding planes whose strength is far weaker than that of the matrix. Shale fracture initiation could occur either in the direction of the maximum tangential stress (*σ*_*ϑϑ*_)_max_ or along the weak bedding surface. Hence, a comprehensive fracture criterion for shale should combine the maximum tangential stress criterion and the weak bedding plane failure criterion together, i.e.,

5 $${({\sigma }_{\vartheta \vartheta })}_{{\rm{\max }}}={\sigma }_{cm},\,{\rm{or}}\,{({\sigma }_{\vartheta \vartheta })}_{\vartheta ={90}^{^\circ }-\beta }={\sigma }_{cb},$$

where (*σ*_*ϑϑ*_) _*ϑ* = 90°−*β*_ is the tangential stress of the bedding plane; *σ*_*cm*_ and *σ*_*cb*_ represent the resistance to fracture of the shale matrix and bedding plane, respectively. Using Eq. (5), two values of failure loads for each orientation can be obtained. During the loading process, the applied load will first reach the smaller value. To express the relative ratio between the stresses of the weak shale bedding plane and the matrix for various bedding plane orientations, a ratio parameter *λ* is defined as follows:

6 $${\lambda }=\frac{{({{\sigma }}_{{\vartheta }{\vartheta }})}_{{\vartheta }=9{0}^{^\circ }-{\beta }}}{{({{\sigma }}_{{\vartheta }{\vartheta }})}_{{\rm{\max }}}}=\frac{{({{\sigma }}^{\ast })}_{{\vartheta }=9{0}^{^\circ }-{\beta }}}{{({{\sigma }}^{\ast })}_{{\rm{\max }}}}.$$

**Figure 11:**
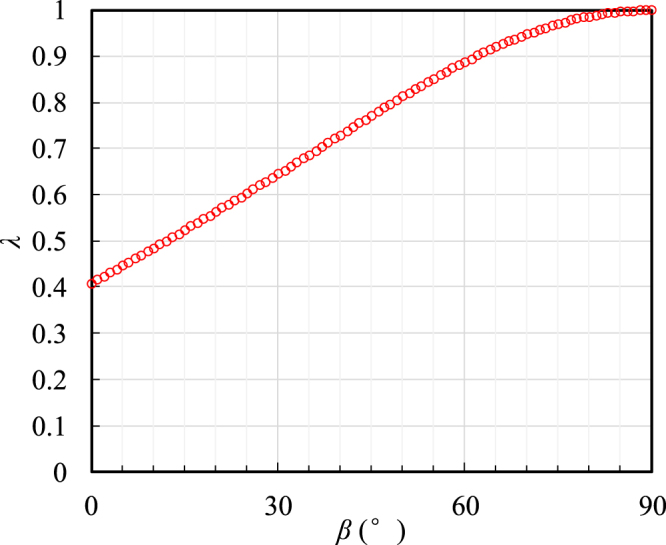
Variations of *λ* with the bedding plane inclination angle *β* for the plane strain case.

Using the measured elastic parameters of the Longmaxi shale, the values of the ratio *λ* for various values of *β* were calculated using Eqs (4) and (6), and the results are shown in Fig. 11. In the figure, *λ* increases with the increase in the bedding plane inclination angle *β*, which implies that the crack propagates more easily along the weak bedding plane when *β* become larger. By synthesizing Eqs (5) and (6), one can determine the criterion for which the crack extends along the bedding plane:

7 $${\lambda }\ge {c}_{0},\,{\rm{and}}\,{c}_{0}={{\sigma }}_{cb}/{{\sigma }}_{cm}.$$

**Figure 12:**
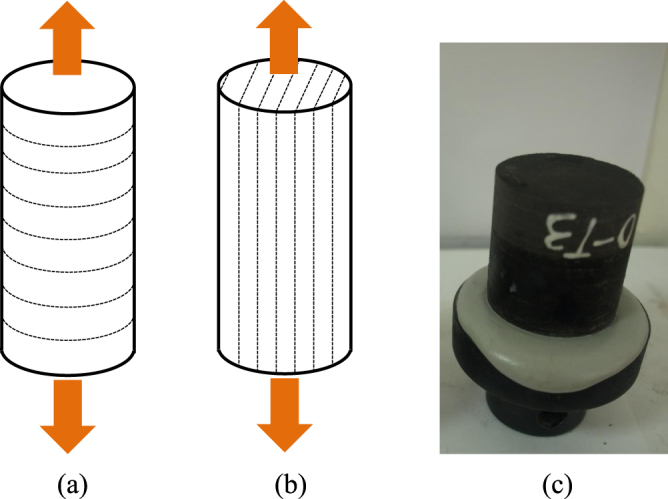
Direct tensile specimens. (**a**) Specimens with loading directions perpendicular to the bedding planes, (**b**) specimens with loading directions parallel to the bedding planes, and (**c**) specimens after direct tensile test.

If the relative tangential stress ratio *λ* cannot meet the above criterion, the crack will propagate in the shale matrix along the direction of the maximum tangential stress. The parameter *λ* is a function of the bedding plane orientation, and the parameter *c*_0_ is a material constant determined by the tensile strengths of the bedding plane and the matrix. The direct tensile strengths of the bedding plane and the matrix can be utilized to characterize their respective abilities to resist crack propagation. To obtain the direct tensile strength of the bedding plane and the matrix, two groups of direct tensile tests for standard core samples, with loading directions either perpendicular or parallel to the bedding planes (Fig. 12), were conducted. Both ends of the core specimen were bonded with steel anchor heads using high strength resin (Fig. 12c). The direct tensile strengths of the samples with a loading direction perpendicular to the bedding planes (Fig. 12a) and the samples with a loading directions parallel to the bedding planes (Fig. 12b) were considered to represent the bedding tensile strength and matrix tensile strength, respectively. The experimental results indicated that the bedding tensile strength was *σ*_*tb*_ = 5.74 MPa and the matrix tensile strength was *σ*_*tm*_ = 8.33 MPa. Substituting the measured values into Eq. (7), one obtains *c*_0_ = 0.6891 for the tested shale. The calculated results for the ratio *λ* for *β* = 0°, 30°, 60° and 90° are 0.4081, 0.6449, 0.8883 and 1, respectively, as shown in Fig. 11. By comparison, when *β* = 0° and 30°, the values of *λ* are less than *c*_0_, corresponding to matrix fracture along the original crack line; while when *β* = 60° and 90°, the values of *λ* are larger than *c*_0_, corresponding to bedding plane failure. The conclusions of this analysis correspond well with our experimental results. The criterion can effectively predict the crack initiation direction when *α* = 0°. Nevertheless, it should be noted that the applicability of the present criterion is limited because the mixed-mode fracture couldn’t be considered, i.e., the criterion is no longer valid after the crack deviation.

## Conclusions

In this study, a series of fracture tests on dry Longmaxi shale with different bedding layer inclination angles was conducted using NDB specimens to further understand the effects of anisotropy on the characteristics of shale fracture toughness and crack extension. The failure loads were measured, and the meso-fracture propagation paths were observed using an SEM. The stress field around a crack tip was analyzed in depth by considering the shale as a transversely isotropic material. The following conclusions were drawn based on the theoretical and experimental studies:

1. For the investigated dry shale NDB specimens with an edge crack along the direction of loading, a crack initiated along the bedding plane when the bedding plane inclination angle was relatively large (*β* = 60° and 90°); in contrast, when the bedding plane inclination angle was small (*β* = 0° and 30°), the fracture penetrated into the matrix.
2. When the crack propagation direction was along the bedding plane, the obtained fracture toughness was lower. The ratio between the maximum *K*_I*c*_ and minimum *K*_I*c*_ was approximately 2.12; this result implies that the anisotropy has a significant effect on the dry shale fracture behavior.
3. The crack tip stress field of anisotropic shale is not only determined by the SIF but also related to the elastic constants and the bedding plane inclination angles.
4. By taking the differences in the strengths of the shale bedding and the matrix into consideration, a criterion for making a preliminary determination regarding whether a crack extends along the bedding plane or penetrates into the matrix was derived.

## Electronic supplementary material

Supplementary Information
