# Supplementary material for: Linear Elastic Fracture Mechanics Characterization of an Anisotropic Shale
Source: Sci Rep. 2018 May 31;8:8505. doi: 10.1038/s41598-018-26846-y (PMC5981205; doi:10.1038/s41598-018-26846-y)
Supplement: Supplementary file 1 — Supplementary Information [file 41598_2018_26846_MOESM1_ESM.pdf]

Linear Elastic Fracture Mechanics Characterization of an Anisotropic Shale

Y. Luo[1] · H.P. Xie[2, 3] · L. Ren[1] · R. Zhang[3] · C.B. Li[1] · C. Gao[1]

1. Key Laboratory of Deep Underground Science and Engineering (MOE), College of Architecture and Environment, Sichuan University, Chengdu 610065, China
2. Institute of Deep Earth Science and Green Energy, Shenzhen University, Shenzhen 518060, China
3. College of Water Resources and Hydropower, Sichuan University, Chengdu 610065, China

Correspondence and requests for materials should be addressed to L.R. (email: renli-scu@hotmail.com)

Appendix

In the plane that is perpendicular to the bedding plane of shale, the relationship between the stress σ_{ij} and the strain ε_{ij} (i, j =1, 2, which correspond to the two principal directions in the shale, respectively) for the plane stress problem can be expressed as[33]

$$\begin{bmatrix} \varepsilon_{11} \\ \varepsilon_{22} \\ 2\varepsilon_{12} \end{bmatrix} = \begin{bmatrix} a_{11} & a_{12} & 0 \\ a_{12} & a_{22} & 0 \\ 0 & 0 & a_{66} \end{bmatrix} \begin{bmatrix} \sigma_{11} \\ \sigma_{22} \\ \sigma_{12} \end{bmatrix}, \tag{1}$$

where a_{ij} represent the flexibility coefficients of the anisotropic material and are defined as

$$a_{11} = \frac{1}{E_1},\ a_{22} = \frac{1}{E_2},\ a_{12} = \frac{-v_{12}}{E_1} = \frac{-v_{21}}{E_2},\ a_{66} = \frac{1}{G_{12}}, \tag{2}$$

where E_1 and E_2 are the Young's moduli in the two principle directions of the shale, G_{12} is the shear modulus in the plane vertical to the bedding plane, and v_{ij} is the Poisson's ratio that defines the strain in the j direction caused by a unit strain in the i direction. For the plane strain case, a_{ij} is replaced by b_{ij}, which can be calculated according to the following equations:

$$b_{ij} = a_{ij} - \frac{a_{i3}a_{j3}}{a_{33}}, \tag{3}$$

where

$$a_{i3} = \frac{-v_{i2}}{E_i} = \frac{-v_{3i}}{E_3},\ a_{33} = \frac{1}{E_3}. \tag{4}$$

where E_3 is the Young's modulus perpendicular to the plane.

For plane symmetric loading, the singular stress and displacement fields at point A in the vicinity of the crack tip in the local Cartesian coordinate system can be expressed analytically as follows[30]:

stresses:

$$
\begin{aligned}
\sigma_{xx} &= \frac{K_{\mathrm{I}}}{\sqrt{2\pi r}}\mathrm{Re}\left[\frac{\mu_1\mu_2}{\mu_1-\mu_2}\left(\frac{\mu_2}{\sqrt{\cos\vartheta+\mu_2\sin\vartheta}}-\frac{\mu_1}{\sqrt{\cos\vartheta+\mu_1\sin\vartheta}}\right)\right] \\
\sigma_{yy} &= \frac{K_{\mathrm{I}}}{\sqrt{2\pi r}}\mathrm{Re}\left[\frac{1}{\mu_1-\mu_2}\left(\frac{\mu_1}{\sqrt{\cos\vartheta+\mu_2\sin\vartheta}}-\frac{\mu_2}{\sqrt{\cos\vartheta+\mu_1\sin\vartheta}}\right)\right], \\
\tau_{xy} &= \frac{K_{\mathrm{I}}}{\sqrt{2\pi r}}\mathrm{Re}\left[\frac{\mu_1\mu_2}{\mu_1-\mu_2}\left(\frac{1}{\sqrt{\cos\vartheta+\mu_1\sin\vartheta}}-\frac{1}{\sqrt{\cos\vartheta+\mu_2\sin\vartheta}}\right)\right]
\end{aligned}
\tag{5}
$$

displacements:

$$
\begin{aligned}
u &= K_{\mathrm{I}}\sqrt{\frac{2r}{\pi}}\mathrm{Re}\left[\frac{1}{\mu_1-\mu_2}\left(\mu_1 p_2\sqrt{\cos\vartheta+\mu_2\sin\vartheta}-\mu_2 p_1\sqrt{\cos\vartheta+\mu_1\sin\vartheta}\right)\right] \\
v &= K_{\mathrm{I}}\sqrt{\frac{2r}{\pi}}\mathrm{Re}\left[\frac{1}{\mu_1-\mu_2}\left(\mu_1 q_2\sqrt{\cos\vartheta+\mu_2\sin\vartheta}-\mu_2 q_1\sqrt{\cos\vartheta+\mu_1\sin\vartheta}\right)\right]
\end{aligned},
\tag{6}
$$

where

$$
\begin{aligned}
p_i &= a'_{11}\mu_i^2 + a'_{12} - a'_{16}\mu_i \\
q_i &= a'_{12}\mu_i + \frac{a'_{22}}{\mu_i} - a'_{26}
\end{aligned},
\tag{7}
$$

in which Re denotes taking the real part of the complex function, and μ_k (k = 1, 2) are related to T_k which are the roots of the characteristic equation for an orthotropic shale material, as determined from the following:

$$
a_{11}T^4 + (2a_{12} + a_{66})T^2 + a_{22} = 0\,,
\tag{8}
$$

where the roots $T_k = \zeta_k + i\eta_k$, and this equation is always true when $\eta_k > 0$. The relationship between μ_k and T_k is expressed by the following formula:

$$
\mu_k = \frac{T_k\cos\varphi + \sin\varphi}{\cos\varphi - T_k\sin\varphi}\,.
\tag{9}
$$

In Eq. (7), a'_{ij} represent the flexibility coefficients in the local x-o-y coordinate system. These coefficients can be obtained from a_{ij} (Eq. (2)) using the following equations:

$$
\begin{aligned}
a_{11}^{'} &= a_{11}\cos^4\varphi + \left(2a_{12}+a_{66}\right)\sin^2\varphi\cos^2\varphi + a_{22}\sin^4\varphi \\
a_{22}^{'} &= a_{11}\sin^4\varphi + \left(2a_{12}+a_{66}\right)\sin^2\varphi\cos^2\varphi + a_{22}\cos^4\varphi \\
a_{12}^{'} &= a_{12} + \left(a_{11}+a_{22}-2a_{12}-a_{66}\right)\sin^2\varphi\cos^2\varphi \\
a_{66}^{'} &= a_{66} + 4\left(a_{11}+a_{22}-2a_{12}-a_{66}\right)\sin^2\varphi\cos^2\varphi \\
a_{16}^{'} &= \left[a_{11}\cos^2\varphi - a_{22}\sin^2\varphi - \left(2a_{12}+a_{66}\right)\cos\left(2\varphi\right)/2\right]\sin\left(2\varphi\right) \\
a_{26}^{'} &= \left[a_{11}\sin^2\varphi - a_{22}\cos^2\varphi + \left(2a_{12}+a_{66}\right)\cos\left(2\varphi\right)/2\right]\sin\left(2\varphi\right)
\end{aligned}
\quad . \qquad (10)
$$
